# Gene expression profiling in non-human primate jejunum, ileum and colon after total-body irradiation: a comparative study of segment-specific molecular and cellular responses

**DOI:** 10.1186/s12864-015-2168-y

**Published:** 2015-11-21

**Authors:** Junying Zheng, Junru Wang, Mylene Pouliot, Simon Authier, Daohong Zhou, David S. Loose, Martin Hauer-Jensen

**Affiliations:** Division of Radiation Health, University of Arkansas for Medical Sciences, Little Rock, Arkansas 72205 USA; CiToxLAB North America, Laval, Quebec Canada H7V 4B3; Integrative Biology and Pharmacology, University of Texas Medical School at Houston, Houston, TX 77030 USA; Surgical Service, Central Arkansas Veterans Healthcare System, Little Rock, Arkansas 72205 USA

**Keywords:** Total body irradiation, Tumor necrosis factor α, Gastrointestinal injury

## Abstract

**Background:**

Although extensive studies have investigated radiation-induced injuries in particular gastrointestinal (GI) segments, a systematic comparison among the different segments on the basis of mode, magnitude and mechanism has not been reported. Here, a comparative study of segment-specific molecular and cellular responses was performed on jejunum, ileum and colon obtained at three time points (4, 7 and 12 days after irradiation) from non-human primate (*Rhesus macaque*) models exposed to 6.7 Gy or 7.4 Gy total body irradiation (TBI).

**Results:**

Pathway analysis on the gene expression profiles identified radiation-induced time-, dose- and segment-dependent activation of tumor necrosis factor α (TNFα) cascade, tight junction, apoptosis, cell cycle control/DNA damage repair and coagulation system signaling. Activation of these signaling pathways suggests that colon sustained the severest mucosal barrier disruption and inflammation, and jejunum the greatest DNA damage, apoptosis and endothelial dysfunction. These more pronounced alterations correlate with the high incidence of macroscopic pathologies that are observed in the colon after TBI. Compared to colon and jejunum, ileum was resistant to radiation injury. In addition to the identification a marked increase of TNFα cascade, this study also identified radiation induced strikingly up-regulated tight junction gene CLDN2 (196-fold after 7.4-Gy TBI), matrix degradation genes such as MMP7 (increased 11- and 41-fold after 6.7-Gy and 7.4-Gy TBI), and anoikis mediated gene EDA2R that mediate mucosal shedding and barrier disruption.

**Conclusions:**

This is the first systematic comparative study of the molecular and cellular responses to radiation injury in jejunum, ileum and colon. The strongest activation of TNFα cascades and the striking up-regulation of its down-stream matrix-dissociated genes suggest that TNFα modulation could be a target for mitigating radiation-induced mucosal barrier disruption.

**Electronic supplementary material:**

The online version of this article (doi:10.1186/s12864-015-2168-y) contains supplementary material, which is available to authorized users.

## Background

Upon total-body irradiation (TBI), high-energy particles penetrate the body and directly or indirectly damage DNA and other key molecular structures within cells. Radiation causes death of rapidly proliferating cells and subsequent alterations that may lead to chronic disorders. The gastrointestinal (GI) system is among the most radiosensitive organs in the body [1, 2]. Acute intestinal radiation injury is marked by massive cell death in the rapidly proliferating crypt epithelium and inflammation in the lamina propria [3–5]. The death of crypt cells results in insufficient replacement of the mucosal epithelium that leads to breakdown of the mucosal barrier and bacterial translocation [6, 7]. In response to injury, the GI system initiates a series of compensated signaling cascades involved in cell death, inflammation, antimicrobial responses and tissue remodelling [8, 9]. Activation of these signaling cascades is a spontaneous protective response of the host that could also exacerbate the destruction of tissues. For example, tumor necrosis factor α (TNFα) promotes an immune response during infection, but excessive and unbalanced TNFα release can directly induce epithelial cell death and cause large-scale mucosal shedding, producing microerosions that cannot be compensated by epithelial tight junction rearrangement [10–13]. In addition, although apoptosis of massively DNA-damaged crypt cells prevents them from reentering the cell cycle [14], the toxins released from dead cells can cause damage to surrounding tissues, which could induce a second wave of cell death [15]. These events occur sequentially after radiation exposure and are governed by underlying cellular and molecular responses.

Because of the distinctive structures and functions of the GI tract segments, differences exist with respect to the cellular and molecular responses of each segment, depending on specific tissue radiosensitivity, damage mode, destruction magnitude and mechanism of injury. Therefore, the different GI segments undergo distinct types of damage after irradiation. Extensive studies have been conducted to investigate radiation-induced injuries in particular segments [5–8]. Small bowel is normally considered the most radiosensitive region due to its rapidly proliferating crypt cells [1, 5]. It is also believed to be a major origin for gut-associated sepsis due to its lack of a thick protective mucous layer [16, 17]. Colon is thought to be more resistant to radiation injury, but symptoms associated with colon damage, such as diarrhea and hematochezia, have been suggested as indicators of survival after a radiation emergency [18]. To our knowledge, a systematic comparison among the different segments with respect to the mode, magnitude and mechanism of TBI-induced injury has not been reported. Understanding the dynamic activation of key signaling pathways and the cellular and molecular responses governing injury can aid in precisely predicting and evaluating the type, mode, magnitude and mechanism of injury, as well as in developing strategies needed to alleviate injuries along the GI tract. Furthermore, the above investigations support characterization of the pathophysiology of the acute radiation injury which is recognized as a regulatory requirement in drug development.

To help understand the mechanisms involved in acute intestinal radiation injury in the different GI tract segments as a basis for developing pharmacological modulators to mitigate the injury, we undertook a study in 24 non-human primate models in which the animals were exposed to 0 Gy, 6.7 Gy or 7.4 Gy TBI. A total of 72 tRNA samples without pooling were collected from jejunum, ileum and colon at three time points (4, 7 and 12 days) after irradiation and subjected to gene expression profiling studies and comparative analysis of the gene expression profiles, including pathway analysis. Activation of signaling cascades suggests that colon undergoes the most severe mucosal barrier interruption and inflammation and that jejunum experiences the most DNA damage and repair, apoptosis and endothelial dysfunction. Compared to jejunum and colon, ileum is the most resistant intestinal segment to radiation injury.

## Results

### Microarray data analysis

The unirradiated jejunum, ileum and colon samples were used as the baseline for gene expression analysis. Genes with significantly changed mRNA levels were selected with a cut-off of *P* < 0.05 and fold change ≥ 2.0. The 18 lists of genes generated from the three segments on days 4, 7 and 12 after 6.7-Gy and 7.4-Gy TBI, including fold-change and P-values, are provided in Additional file 1: Table S1; Table 1 shows the 18 lists of genes that are in that file.

**Table 1:** Summary of 18 lists of genes altered ≥ 2.0-fold from jejunum, ileum and colon after exposure to 6.7-Gy and 7.4-Gy TBI. Available in Additional file 1

Time points	Jejunum	Ileum	Colon
6.7 Gy_4d	767 (478-up, 289-down)	256 (141-up, 115-down)	1306 (640-up, 666-down)
6.7 Gy_7d	561 (206-up, 355-down)	208 (71-up, 137-down)	420 (196-up, 224-down)
6.7 Gy_12d	409 (155-up, 254-down)	622 (362-up, 260-down)	454 (278-up, 176-down)
7.4 Gy_4d	638 (456-up, 182-down)	743 (468-up, 275-down)	2574 (1263-up, 1311-down)
7.4 Gy_7d	417 (162-up, 255-down)	261 (71-up, 190-down)	104 (47-up, 57-down)
7.4 Gy_12d	488 (248-up, 240-down)	520 (226-up, 294-down)	539 (366-up, 173-down)

Note: The number outside the parenthesis is the total number of genes with mRNA level altered ≥ 2.0-fold. Number in the parenthesis is the number genes with mRNA level up-regulated or down-regulated ≥ 2.0-fold

### Verification of gene expression by RT-qPCR

**Fig. 1:**
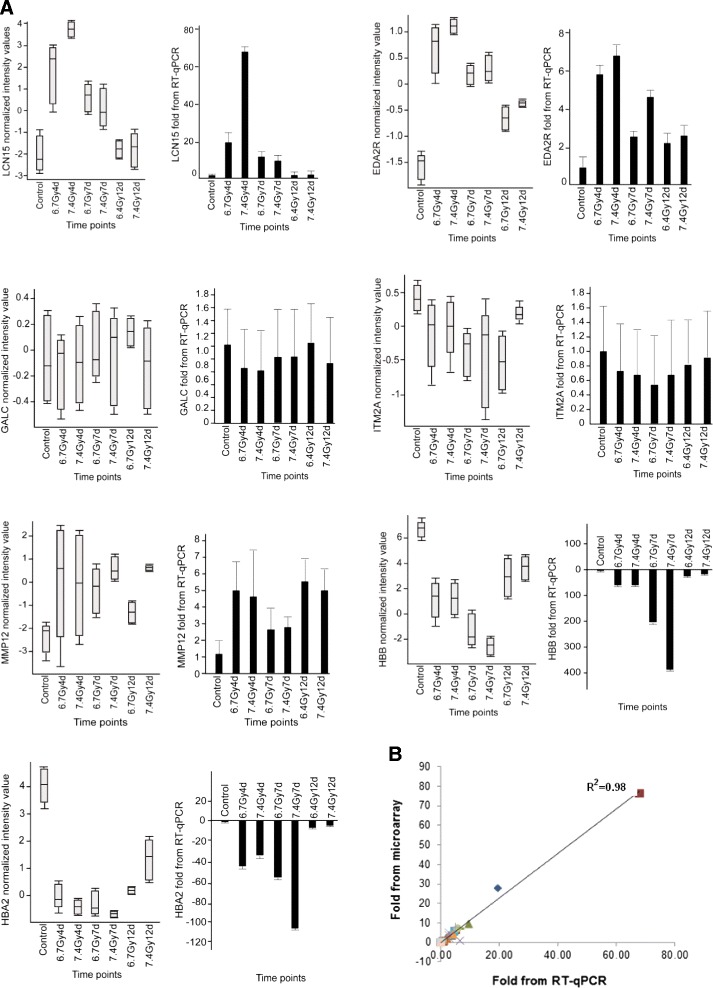
Validation of selected genes in colon by real-time RT-qPCR (**a** and **b**). Expression of the selected genes at the different time points after TBI was plotted by microarray normalized signals (grey bars). The relative expression of the same selected genes was normalized by GAPDH gene at the different time points by real-time RT-qPCR (black bars). The opposite direction of HBB and HBA2 demonstrates their down-regulation after radiation (panel **a**). Statistics analysis was performed to estimate the consistency between RT-qPCR results and the microarray data (panel **b**)

Colon segments were selected for microarray validation. The expression of LCN15, EDA2R, HBB, HBA2, MMP12, GALC and ITM2A in control and samples irradiated at the different time points after 6.7-Gy and 7.4-Gy TBI was validated by real-time RT-qPCR. In Fig. 1a, the expression of the selected transcripts at the different time points is plotted by normalized signals from the microarray (grey bars), together with their relative expression after normalization by GAPDH (black bars) in RT-qPCR. Figure 1b demonstrates a statistics analysis to estimate the consistency between RT-qPCR results and the microarray data.

### Ingenuity pathway analysis (IPA) upstream regulator analysis

**Fig. 2:**
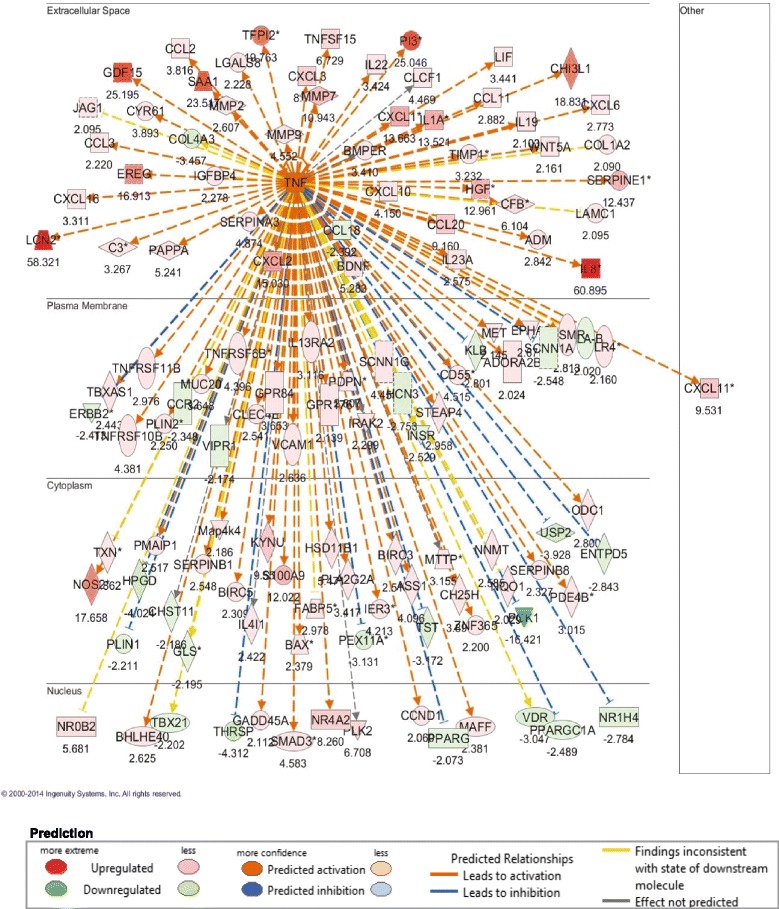
TBI at 6.7 Gy induced significant activation of TNFα cascades in colon tissue at day 4 (z-score, 6.7; P-value = 3.85e-23). An absolute z-score of ≥ 2 is considered significant

**Fig. 3:**
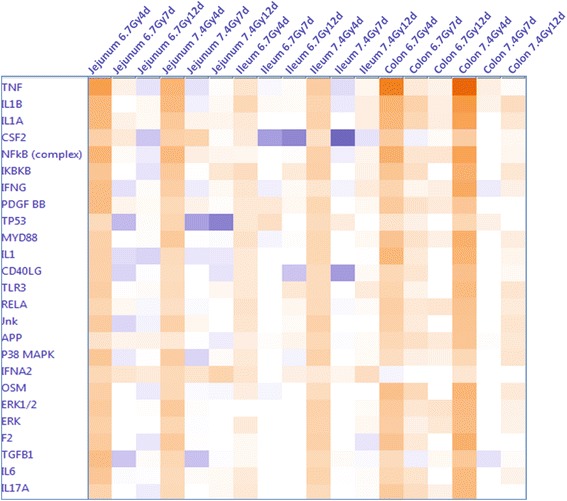
Comparative analysis of the dynamic activation of upstream regulators in jejunum, ileum and colon after 6.7 Gy and 7.4 Gy TBI. An absolute z-score of ≥ 2 is considered significant. An upstream regulator was considered increased (activated) if the z-score was ≥ 2 (orange), and decreased (inhibited) if the z-score was ≤ −2 (blue)

IPA Upstream Regulator Analysis revealed that TBI induced extensive activation of TNFα cascades in colon at day 4 (Fig. 2). Activation Z-score was calculated as a measure of functional and translational activation in Networks and Upstream Regulators Analysis. A Z-score of < −2 (inhibited) or > 2 (activated) was considered as significant. Table 2 shows the Z-scores of each segment that predict TNFα activation in jejunum, ileum and colon after 6.7-Gy and 7.4-Gy TBI. TNFα cascades were strongly activated in all segments at day 4. Activation of TNFα was dose- and segment-dependent, with colon showing the strongest activation after 7.4-Gy TBI with a Z-score 9.3 and ileum the least activation after 6.7-Gy TBI with a Z-score 2.29. Activation of TNFα cascades were significantly suppressed on days 7 and 12 in all three gut regions (Table 2). In an unbiased analysis of activation of all of the upstream regulators, the top activated upstream regulators, in addition to TNFα, included interleukin 1B and 1A (IL1B and IL1A), with colon being the most activated and ileum the least activated segment (Fig. 3).

**Table 2:** Dynamic activation Z-scores^*a*^ of TNFα cascades in jejunum, ileum and colon at days 4, 7, and 12 after 6.7 Gy and 7.4 Gy TBI

Time points (d)	Z-score (Jejunum)		Z-score (Ileum)		Z-score (Colon)	
	6.7 Gy	7.4 Gy	6.7 Gy	7.4 Gy	6.7 Gy	7.4 Gy
4	6.46	5.32	2.29	3.8	7.9	9.3
7	1.26	−0.89	−0.31	−1.06	1.9	1.39
12	−0.88	0.29	0.38	0.89	1.26	0.45

^*a*^An absolute Z-score of ≥ 2 is considered significant. An upstream regulator is predicted to be activated if the Z-score is ≥ 2 and inhibited if the Z-score ≤ −2

### IPA canonical pathway analysis

#### Activation of tight junction signaling in jejunum, ileum and colon

**Fig. 4:**
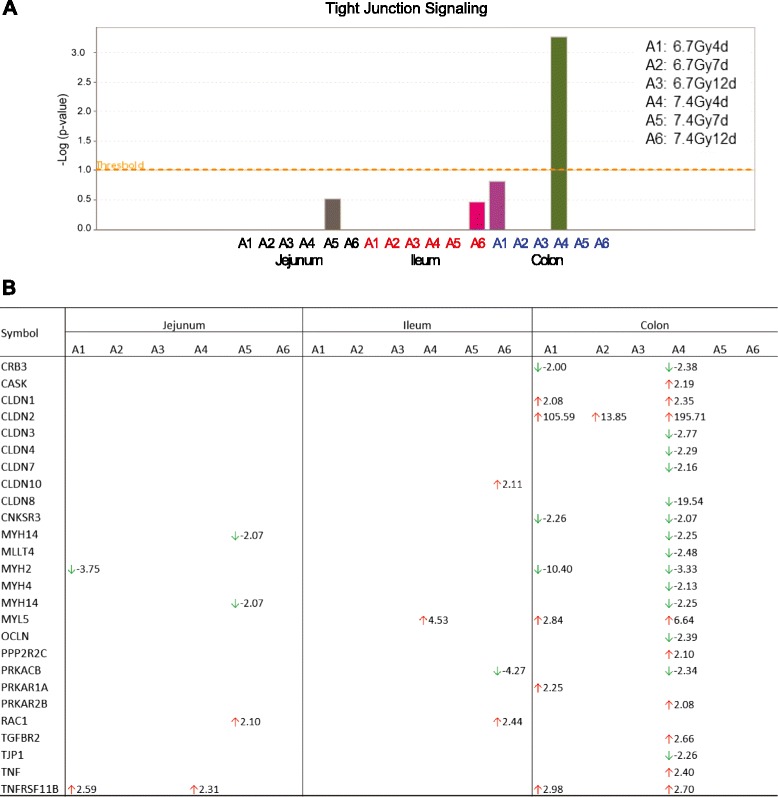
Comparative analysis demonstrates that the tight junction signaling pathway is only activated in colon on day 4 after 7.4 Gy TBI (panel **a**). Pathways with a –log value (*P*-value) above the threshold (dashed line) were significantly activated. Altered transcripts involved in tight junction signaling in jejunum, ileum and colon at the different time points after 6.7 Gy and 7.4 Gy TBI (panel **b**). The font color of A1 to A6 represents segment of jejunum (black), ileum (red) and colon (blue). A1 to A6 represent the time point of days 4, 7 and 12 after 6.7 Gy and 7.4 Gy TBI

As epithelial tight junctions play a key role in intestinal mucosal permeability (25, 26), we investigated the expression of tight junction–associated genes in the different GI segments after irradiation. The lists of transcripts altered ≥ 2-fold at each time point were imported into IPA to identify the activation of tight junction signaling pathways. Analysis of the gene expression profiles revealed that tight junction signaling was only significantly activated in colon on day 4 after 7.4-Gy TBI; there was no significant activation in jejunum or ileum or at the other time points in colon (Fig. 4a). Figure 4b lists the altered genes corresponding to the tight junction pathway. Radiation induced very few altered genes in jejunum and ileum. However, 7.4Gy TBI induced 23 genes with mRNA level altered ≥ 2-fold in colon at day 4. Of these genes, CLDN2 was strikingly increased 105- and 196-fold after 6.7-Gy and 7.4-Gy TBI, respectively. Expression of CLDN3, CLDN4, CLDN7 and CLDN8 were all significantly down-regulated. All of these changes were substantially attenuated on days 7 and 12.

#### Activation of immune response pathways in jejunum, ileum and colon

**Fig. 5:**
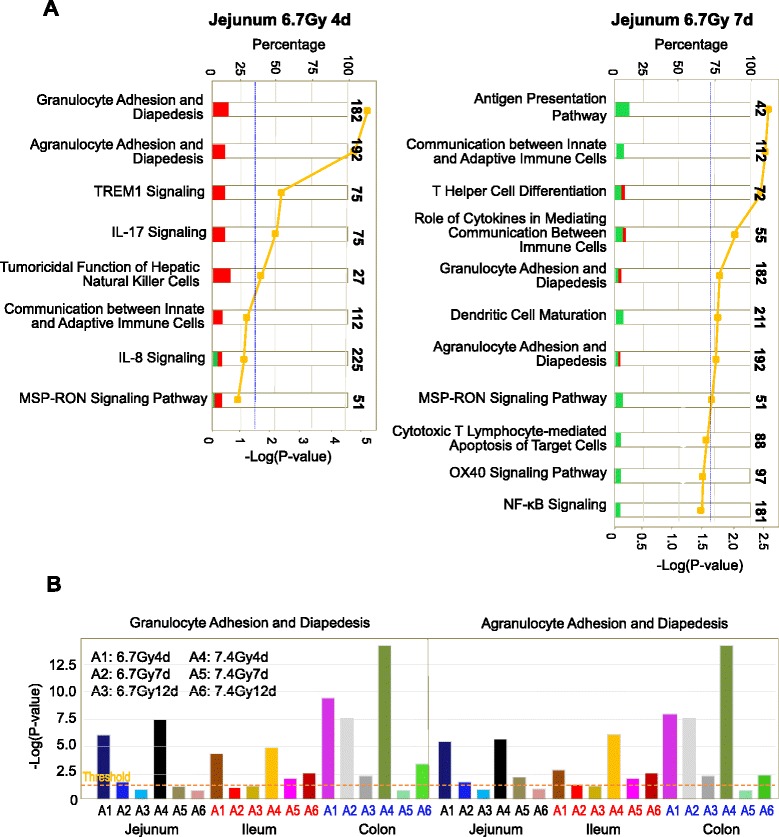
The various immune response signaling pathways activated in jejunum on day 4 after 6.7 Gy TBI were significantly suppressed by day 7. Stacked bar charts demonstrate IPA-generated activated pathways after irradiation. The width of a bar indicates the percentage of transcripts that changed in the particular pathway (red bar: up-regulated; green bar: down-regulated). Pathways with a P-value (yellow square) above the threshold (dashed line) were significantly regulated (panel **a**). A comparative analysis of granulocyte and agranulocyte adhesion signaling and diapedesis in jejunum, ileum and colon on days 4, 7 and 12 after 6.7 Gy and 7.4 Gy TBI revealed pathways that were significantly activated above the threshold –Log value (P-value) (dashed line) (panel **b**)

The analysis by IPA of activation of immune response pathways in jejunum, ileum and colon showed that TBI induced a wide activation of these pathways in all three intestinal segments at day 4, with significant suppression of the activated pathways at days 7 and 12 (Fig. 5). The activation was dose- and region-dependent, with colon exposed to 7.4-Gy TBI showing the highest degree of pathway activation and ileum exposed to 6.7-Gy TBI showing the least activation. Various immune response pathways in jejunum were activated on day 4 after 6.7-Gy TBI and were suppressed at day 7 (Fig. 5a). A comparative analysis of granulocyte adhesion and agranulocyte adhesion signaling in relation to diapedesis in jejunum, ileum and colon on days 4, 7 and 12 after 6.7-Gy and 7.4-Gy TBI identified pathways significantly activated above the threshold –Log value (*P*-value) (Fig. 5b, dashed line). Granulocyte and agranulocyte adhesion signaling and diapedesis were significantly activated at day 4 in all three segments, with colon the most activated site. These activations were significantly suppressed at days 7 and 12. The altered genes regulating granulocyte adhesion and diapedesis pathways in jejunum, ileum and colon at days 4, 7 and 12 after 6.7-Gy and 7.4-Gy TBI are shown in Additional file 2: Table S2.

#### Activation of aryl hydrocarbon receptor (AhR)-mediated apoptosis in jejunum, ileum and colon

**Fig. 6:**
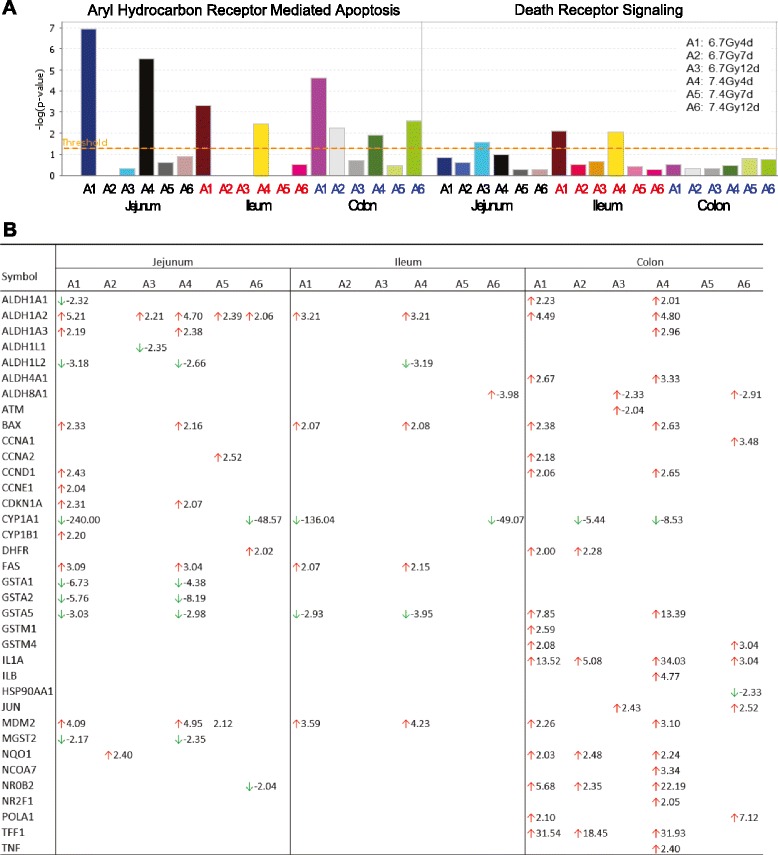
TBI induced significant activation of the AhR-mediated apoptosis signaling pathway in jejunum, ileum and colon at the early time point of 4 days after TBI. Pathways with a –log value (P-value) above the threshold (dashed line) were significantly activated (panel **a**). Genes involved in AhR signaling altered at the different time points after 6.7 Gy and 7.4 Gy TBI (panel **b**)

TBI induced significant activation of AhR-mediated apoptosis signaling pathway. AhR was strongly activated at day 4 after both TBI doses in all intestinal segments, with jejunum the most activated segment, and was significantly suppressed or attenuated in all segments at days 7 and 12 (Fig. 6a). Figure 6b lists the altered genes corresponding to the AhR signaling pathway. TBI also activated other apoptotic signaling pathways, such as death receptor signaling (Fig. 6a); however, the magnitude of activation of these pathways was much lower than that of the AhR-mediated apoptosis pathway.

#### Activation of cell cycle signaling pathways in jejunum, ileum and colon

**Fig. 7:**
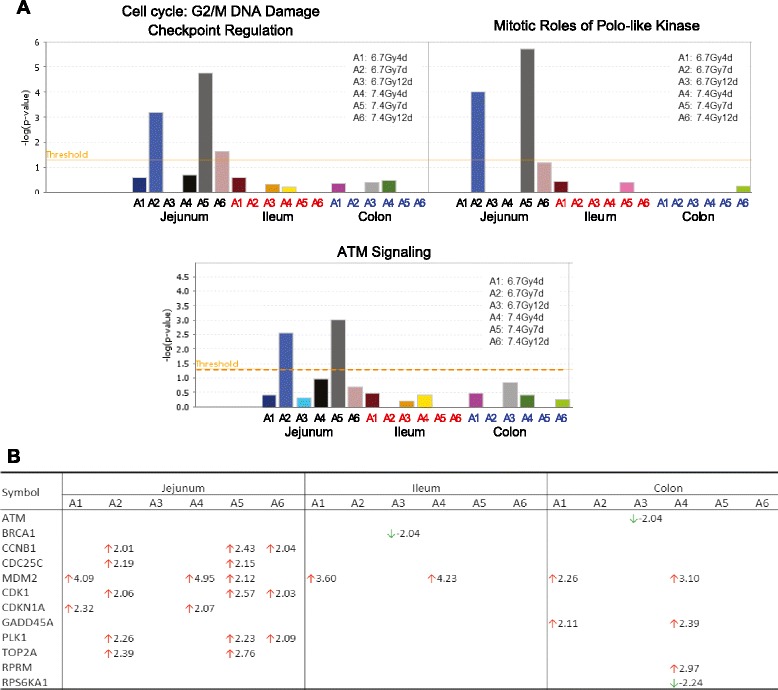
Activation of cell cycle control and DNA damage repair signaling pathway in jejunum, ileum and colon after TBI. Pathways with a –log value (*P*-value) above the threshold (dashed line) were significantly activated (panel **a**). Genes involved in G2/M DNA damage checkpoint regulation altered at the different time points after 6.7 Gy and 7.4 Gy TBI (panel **b**)

In response to radiation-induced DNA damage, pathways regulating cell cycle control and DNA damage repair signaling were activated. Activation of pathways regulating the G2/M DNA damage checkpoint regulation, mitotic roles of Polo-like kinases, and ATM signaling in jejunum was significant at day 7 at both TBI doses and was significantly suppressed at day 12 (Fig. 7). No significant activation of these pathways was observed in ileum or colon. Figure 7b lists the genes whose expression were altered involved in the G2/M DNA damage checkpoint regulation pathway.

#### Activation of coagulation system in jejunum, ileum and colon

**Fig. 8:**
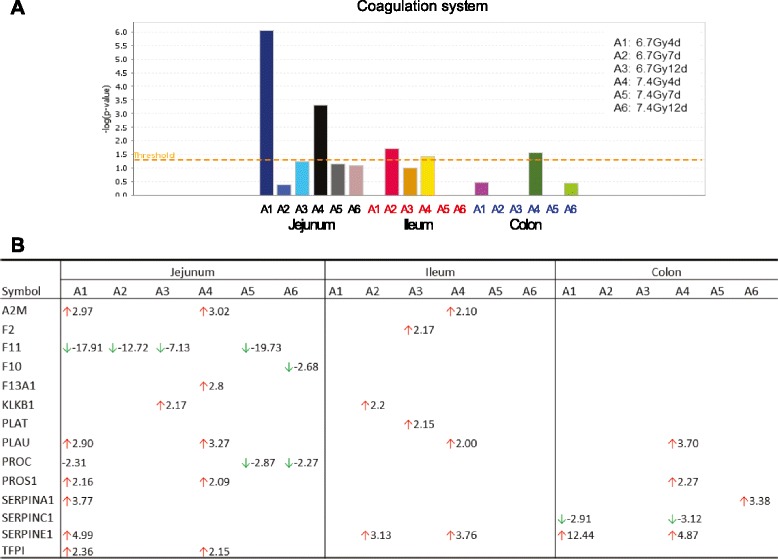
TBI induced significant activation of the coagulation system in jejunum, but not in ileum or colon. Pathways with a –log value (*P*-value) above the threshold (dashed line) were significantly regulated (panel **a**). Genes involved in the coagulation system altered at the different time points after 6.7 Gy and 7.4 Gy TBI (panel **b**)

Analysis of cellular stress and injury signaling identified significant activation of the coagulation system in jejunum at day 4 after irradiation at both TBI doses, with complete suppression at days 7 and 12 (Fig. 8). Activation of the coagulation system was marginally significant in ileum or colon. Figure 8b lists the genes whose expression were altered involved in the coagulation system.

#### Up-regulation of anoikis signaling and TNF-mediated matrix-dissociated genes

**Fig. 9:**
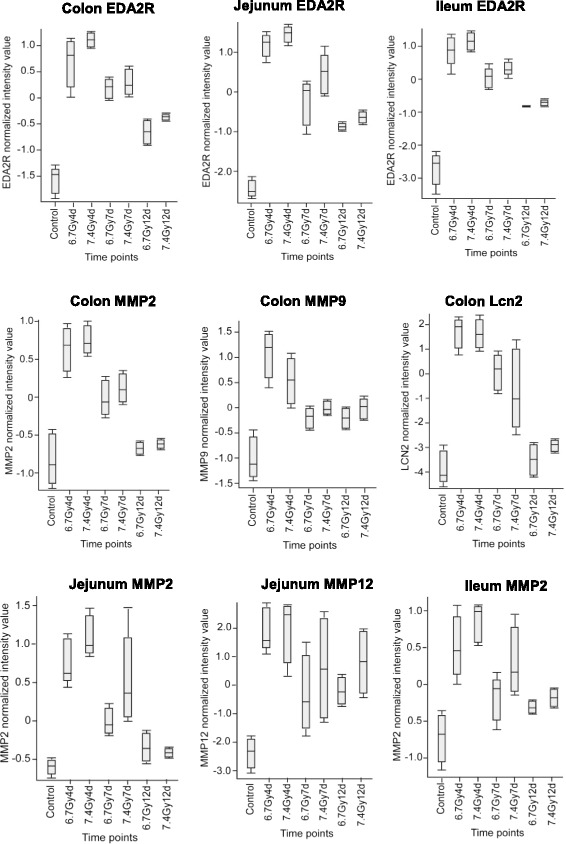
Up-regulation of EDA2R and matrix-dissociated transcripts in jejunum, ileum and colon after 6.7 Gy and 7.4 Gy TBI

TBI induced significant up-regulation of EDA2R in all three segments after both radiation doses. EDA2R is a type III transmembrane protein of the TNF receptor superfamily that is associated with mediating anoikis, The increase of EDA2R mRNA expression reached a maximum at day 4 and gradually returned toward the baseline at days 7 and 12 (Fig. 9). Radiation also induced marked up-regulation of various matrix metalloproteinases (MMPs). The up-regulated MMPs in colon include MMP2, MMP7, MMP9 and MMP12 (Figs. 9 and Additional file 1: Table S1) and in jejunum MMP2 and MMP12. In ileum, only MMP2 was significantly induced. In addition to various types of MMPs, we identified a striking increase of LCN2 in colon at day 4 (58-fold increase after 6.7-Gy TBI) (Fig. 2), which was significantly suppressed at day 12. The striking increase of LCN2 only occurred in colon, not in jejunum and ileum (Additional file 1: Table S1). IPA upstream analysis showed that these induced MMP genes and LCN2 were directly activated by TNFα (Fig. 2).

### Immunohistochemistry for TNFα

**Fig. 10:**
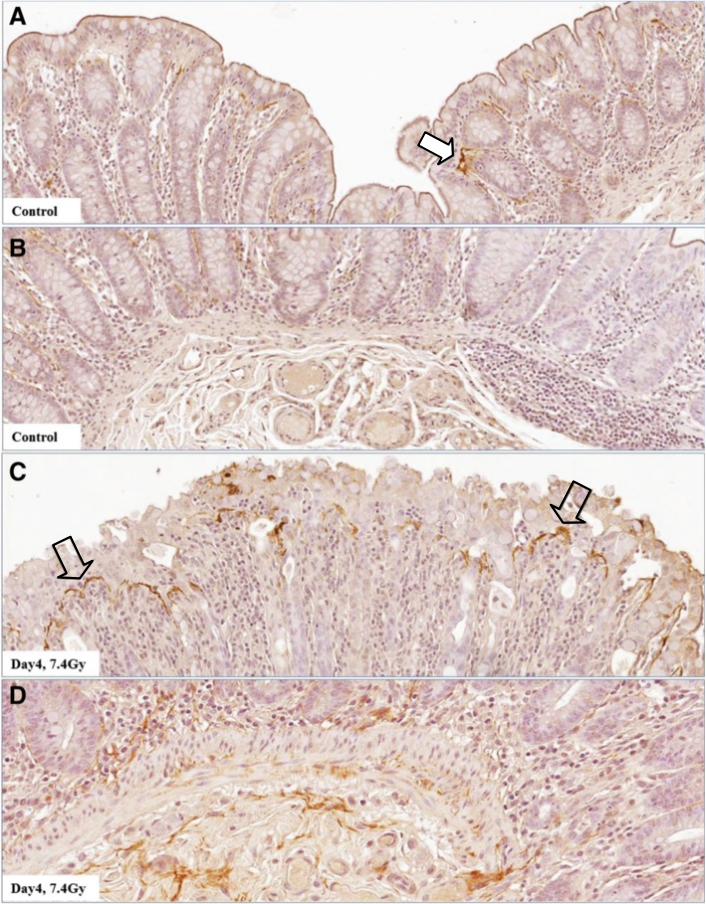
Immunohistochemistry in colon tissue sections for TNFα protein. In unirradiated colon, TNFα protein is weakly distributed across the section; however, a streak of some visible signals can be seen beneath the intact mucosal epithelial lining (panels **a** and **b**, solid white arrow). At day 4 after 7.4-Gy TBI, TNFα staining in the interstitial space from serosa to mucosa and submucosa of the colon exhibits significantly increased TNFα staining. Note the strong streak of TNFα staining in the mucosal region close to the epithelial barrier, forming an interface between swollen/disrupted cells and normal-shaped cells (panels **c** and **d**, open arrows)

Immunohistochemistry was performed on colon tissue sections from control and irradiated samples collected on study day 4 after 7.4-Gy TBI. Staining for TNFα was very weak across the normal colon section, except for some visible signals distributed as a streak underneath the intact mucosal epithelial lining (Fig. 10a, b). In the irradiated colon section, a significant increase in TNFα staining was observed in the mucosa, submucosa and serosa (Fig. 10c, d). In the mucosal region close to the epithelial barrier, TNFα had accumulated as a strong streak that formed an interface between swollen/disrupted cells and normal-shaped cells (Fig. 10c).

## Discussion

This comparative analysis of the gene expression profiles of jejunum, ileum and colon obtained at three time points (4, 7 and 12 days) from *Rhesus macaque* models exposed to 6.7 Gy and 7.4 Gy TBI has yielded valuable insights into the differences in the radiosensitivity of these segments, as well as differences with respect to damage mode, destruction magnitude, and mechanism of injury. Pathway analysis of the gene expression profiles identified radiation-induced time-, dose- and segment-dependent activation of TNFα cascade, tight junction, apoptosis, cell cycle control/DNA damage repair and coagulation system signaling. On the basis of the activation of these signaling cascades and pathways, colon sustained the most severe mucosal barrier interruption and inflammation, and jejunum the most DNA damage, apoptosis and endothelial dysfunction. Compared to colon and jejunum, ileum was the most resistant segment to radiation exposure.

The TNFα cascade was identified as the upstream regulator of gene expression profile changes after TBI with the highest activation score as defined by IPA Upstream Regulator analysis. Activation of TNFα differed by intestinal segment, with colon showing the highest level of activation and ileum the lowest. TNFα cascade activation was also time-dependent, occurring at day 4 and then being completely suppressed at days 7 and 12. TNFα is a pro-inflammatory molecule that regulates multiple cellular processes [9]. In intestine, it regulates inflammation, mucus secretion, cell survival and death [9–13]. Its protective or destructive role primarily depends on the dosage and activation of secondary signaling pathways. For example, TNFα overexpression by exogenous administration or specific knock-in induces abnormal epithelial shedding producing microerosions that cannot be sealed by tight junction rearrangement [19]. Clinically, many studies have implicated TNFα’s intricate link to intestinal barrier disruption in inflammatory bowel diseases such as Crohn’s disease and ulcerative colitis [9]. In irradiated colon, where the TNFα cascade was highly activated, histology revealed large-scale epithelial shedding with more severe barrier disruption than in other GI segments.

The distribution pattern of immunohistochemical staining for TNFα in irradiated colon demonstrates a direct involvement of TNFα in radiation-induced mucosal shedding. At day 4 after 7.4 Gy TBI, the interstitial space from serosa to mucosa showed a marked increase of TNFα staining, and, in an intriguing finding, a strong streak representing accumulation of TNFα was observed in the mucosal region close to the epithelial barrier, separating abnormally from normally shaped cells (see Fig. 10). Regulation of intestinal inflammation and mucosal injury by TNFα has undergone extensive study. Targeting of TNFα with antibodies has been successfully used in the clinical treatment of inflammatory bowel disease for many years, highlighting the importance and clinical relevance of TNFα’s function in intestinal inflammation [20]. Our finding in normal colon of a visible streak of TNFα closely adjacent to the base of the epithelial lining (see Fig. 10a, b) suggests that TNFα could act as a sentinel in the response to barrier disruption.

Of the TNFα downstream targets, genes regulating cellular dissociation including MMP2, MMP3, MMP7, MMP9 and LCN2 were strikingly increased in colon tissue after 7.4 Gy irradiation. Among these in colon, MMP7 was increased 11- and 41-fold after 6.7 and 7.4 Gy TBI, and LCN2 was increased 58- and 52-fold, respectively. Epithelial MMP-7 breaks down extracellular matrix (ECM) and activates α-defensin produced by paneth cells [21]. LCN2 protein, also called NGAL (neutrophil gelatinase–associated lipocalin), can form a complex with MMP-9 and strongly enhance and preserve MMP-9 activity [22]. The strikingly increased expression of these matrix degradation genes inevitably causes cells to lose their attachment to the surrounding ECM. In addition, radiation induced strong expression of EDA2R, a member of the TNF receptor family that mediates p53-regulated anoikis [23]. Anoikis is a form of programmed cell death induced by detachment of anchorage-dependent cells from the surrounding ECM. EDA2R protein negatively regulates focal adhesion kinase, a central component of focal adhesion. In humans, activated EDA2R gives rise to a clinical syndrome characterized by loss of hair, sweat glands and teeth [24]. We hypothesize that activation of the EDA2R and TNFα cascades plays a key role in radiation-induced mucosal shedding in colon. Interestingly, these matrix degradation genes showed a much lower increase in jejunum and ileum than in colon.

Intestinal mucosal barrier disruption in response to TBI changes the barrier’s permeability. Our investigation of the expression of epithelial tight junction–associated genes in the different GI segments after irradiation revealed that only colon exposed to 7.4 Gy TBI caused significant (≥2-fold) changes in numerous tight junction genes at day 4, with very few alterations in expression of tight junction–associated genes observed in jejunum and ileum (see Fig. 4a). In intestine, tight junction proteins form a barrier against unlimited paracellular passage of solutes and water; they also form channels allowing distinct permeation across the barrier [25, 26]. Among the tight junction–associated genes that were up-regulated after irradiation was CLDN2, which showed striking 106- and 196-fold increases after 6.7 Gy and 7.4 Gy TBI, respectively. CLDN2 protein (claudin 2) has been shown to be of great clinical impact. It is a predominant pore-forming protein for selective transportation of water and small cations (e.g., Na + and K+) [27–29]. An increase of CLDN2 protein expression causes excessive diffusion of ions and water from blood to lumen, leading to leak-flux diarrhea [27, 28]. CLDN2 expression can be induced by TNFα and has been reported in various diseases, including Crohn’s disease, ulcerative colitis and celiac disease [27, 28]. In patients with Crohn’s disease, the expression of CLDN2 is dramatically up-regulated, especially in patients with low expression of the sealing tight junction protein CLDN8 (claudin 8) [28]. In our studies, up-regulated expression of CLDN2 after 7.4 Gy TBI was also accompanied with a wide down-regulation of sealing tight junction genes, including CLDN8, which was down-regulated 20-fold. These data suggest a direct link between tight junction disruption and the diarrhea observed in most of the irradiated animals from days 2 to 7.

Activation of the TNFα cascade and tight junction signaling suggests that colon suffered the severest mucosal barrier disruption after TBI. This conclusion is further supported by the activation of immune response signaling pathways observed among the segments. Breakdown of the mucosal barrier causes bacterial translocation into the deep gut tissue and triggers an immune response and inflammation. Activation of these signaling pathways enables the organism to defend itself against infectious microbes. The magnitude of activation reflects the degree of tissue injury. Among the segments studied, colon had the greatest number of altered immune response transcripts and the highest magnitude of change. For example, the IL1A gene was increased 14- and 34-fold in colon at day 4 after 6.7 Gy and 7.4 Gy TBI, respectively, but was not induced in jejunum and ileum. The same was the case with CCL11, CCL23, CXCL16, CXCL17, MMP9 and CLDN2. These transcripts were markedly up-regulated in colon but not induced in jejunum and ileum. In addition, CXCL8 was increased 61- and 56-fold in colon at day 4 after 6.7-Gy and 7.4-Gy TBI, respectively, but only 7- and 4-fold in jejunum and ileum after the higher-dose TBI. These data seem reasonable in view of the disruption of the epithelial barrier in colon, the largest reservoir of microorganisms [30, 31], which would prompt a strong defensive reaction to fight against the invading pathogens. As was the case with activation of the TNFα cascade and tight junction signaling, activation of the immune response was time-dependent, with day 4 as the most activated time point. At days 7 and 12, the activated immune response pathways were significantly suppressed.

In contrast to the colon, where TNFα cascade, tight junction and immune response signaling were most highly activated; jejunum was the segment with the strongest activation of apoptosis, cell cycle control/DNA damage repair and coagulation system signaling after irradiation. After radiation exposure, a wave of apoptosis normally occurs within a few hours. But in this study, we found significant activation of AhR-mediated apoptotic signaling at day 4 after both doses of TBI, with jejunum the most activated segment. AhR was originally characterized as a regulator of xenobiotic metabolism and currently is considered an important mediator of toxic responses [32, 33]. Activation of AhR signaling could be associated with the second wave of apoptosis induced by the toxicity generated by reactive oxygen species, ruptured cells or invasive microorganisms. TBI induced a wide activation of pathways that regulate cell cycle control and DNA damage repair [34, 35]. Surprisingly, activation of various DNA damage repair pathways occurred in jejunum, not in ileum or colon. The lack of DNA damage repair might explain why colon has a higher incidence of adenocarcinoma [36]. Previous studies, including ultrastructural studies, have shown that endothelial cells are also among the most radiation-sensitive cell types [37, 38]. Radiation induced a significant activation of coagulation system signaling in jejunum at day 4. The strongest activation of the coagulation system in jejunum, but not ileum or colon, could be due to the abundance of blood capillaries in jejunal villi for nutrient absorption [39, 40].

## Conclusions

This is the first systematic comparative study of the molecular and cellular responses to radiation injury in jejunum, ileum and colon. Activation of signaling cascades suggests that colon undergoes the most severe mucosal barrier interruption and inflammation and that jejunum experiences the most DNA damage and repair, apoptosis and endothelial dysfunction. These more pronounced alterations correlate with the high incidence of macroscopic pathologies that are observed in the colon after total body irradiation. Compared to jejunum and colon, ileum is the most resistant intestinal segment to radiation injury. The strongest activation of TNFα cascades and the striking up-regulation of its down-stream matrix-dissociated genes suggest that TNFα modulation could be a target for mitigating radiation-induced mucosal barrier disruption. Future studies will verify these findings at the post-transcriptional and post-translational levels.

## Methods

### Animal model, intestinal tissue collection and total RNA preparation

Procedures involving the care and use of animals in this study were reviewed and approved by the Institutional Animal Care and Use Committee (IACUC) prior to conducting the experiments. All procedures were conducted according to the Guide for the Care and Use of Laboratory Animals (8th Ed., National Academy of Sciences) in the Association for Assessment and Accreditation of Laboratory Animal Care (AAALAC) accredited facility. Veterinary cares were available 24 h/7d throughout the study. The animal room environment was controlled (temperature 21 ± 3 °C, humidity 30–70 %, 12 h light, 12 h dark, 10–15 air changes per h) and temperature and relative humidity were monitored continuously. A standard certified commercial primate chow (Certified Primate Diet 2055CTM, Harlan Teklad, Madison, WI, USA) was available to each animal twice daily. Enrichment included fruits and other treats, television and radio.

At total of 24 young-adult male *Rhesus macaque* primates without known preexisting disease underwent sham-irradiation or exposure to a single uniform total-body dose of 6.7-Gy (LD70/30) or 7.4-Gy (LD90/30) of gamma radiation from a cobalt-60 source (Theratron 1000, Best Theratronics, Ottawa, Ontario, Canada). The duration of the observation period before euthanasia was 4, 7 and 12 days following TBI. Intestinal tissues from jejunum, ileum and colon were collected from unirradiated control (*n* = 4) and irradiated animals on days 4 (*n* = 4), 7 (*n* = 4), and 12 (*n* = 2) after 6.7-Gy and 7.4-Gy TBI and stored in RNAlater. The same unirradiated controls (*n* = 4) were used for both 6.7-Gy and 7.4-Gy exposures. The length of each segment is around 3 cm and whole gut tissue (~50 mg) was used for tRNA extraction.

Total RNA was extracted with the use of RNeasy microarray tissue mini kits (Qiagen, Germantown, MD) and cleaned by TURBO DNase (Life Technologies/Thermo Fisher Scientific, Grand Island, NY). The purity of RNA (OD260/280, OD260/230) was measured by NanoDrop 2000c spectrophotometer (Thermo Fisher Scientific, Waltham MA). RNA integrity was evaluated on a Bioanalyzer 2100 (Agilent Technologies, Santa Clara, CA). RNA integrity number (RIN) values below 6 were considered failed and not sent for microarray processing.

### Verification of gene expression by RT-qPCR

Gene expression was verified with the same sets of total RNA samples sent for microarray. For each RNA sample, 2 μg was used as the template for cDNA synthesis by using the High-Capacity cDNA reverse transcription kit (Life Technologies/Thermo Fisher Scientific). Steady-state mRNA levels were measured with real-time quantitative RT-qPCR using the following predesigned TaqMan (Life Technologies/Thermo Fisher Scientific) gene expression assays for the following genes: LCN15, ITM2A, EDA2R, MMP12, HBB, HBA2 and GALC. PCR amplification and detection were carried out on an ABI Prism 7000 sequence detection system (Life Technologies/Thermo Fisher Scientific). Transcript levels were normalized to GAPDH and calculated relative to unirradiated controls by the standard ΔΔCt method. A statistics analysis was performed to estimate the consistency between RT-qPCR results and the microarray data.

### Gene microarray and data analysis

A total 72 RNA samples (Table 3) extracted from jejunum, ileum and colon from un-irradiated control (*n* = 4) and irradiated animals on days 4 (*n* = 4), 7 (*n* = 4), and 12 (*n* = 2) after 6.7-Gy and 7.4-Gy TBI were processed for gene microarray analysis without pooling. For each sample, 150 ng of total RNA was converted to cDNA, amplified and labelled using the Low input quick amp one-color labelling kits from Agilent following the manufactures protocol. A total of 1.65 μg of labelled cDNA was hybridized to Agilent single-color Rhesus Macaque (V2) gene expression 4 x 44 K slides (Agilent Technologies,). Arrays were washed, and data were analysed with Agilent’s GeneSpring GX 11.0 software. Briefly, raw data were log2-transformed and then normalized to the 75th percentile of all values on a chip. Samples from unirradiated jejunum, ileum and colon were used as the baseline controls.

**Table 3:** Summary of 72 tRNA samples processed for microarray without pooling

Segments dose	Control	4 days	7 days	12 days
Jejunum 6.7Gy	*N* = 4	*N* = 4	*N* = 4	*N* = 2
Jejunum 7.4Gy		*N* = 4	*N* = 4	*N* = 2
Ileum 6.7Gy	*N* = 4	*N* = 4	*N* = 4	*N* = 2
Ileum 7.4Gy		*N* = 4	*N* = 4	*N* = 2
Colon 6.7Gy	*N* = 4	*N* = 4	*N* = 4	*N* = 2
Colon 7.4Gy		*N* = 4	*N* = 4	*N* = 2

### Pathway analysis

All transcripts that were differentially expressed more than 2-fold at P < 0.05 between TBI and control at each time point (Additional file 1: Table S1) were imported into Ingenuity Pathway Analysis (IPA) software (Qiagen/Ingenuity, Redwood City, CA) for pathway analysis. Upstream Regulator Analysis in IPA was performed to identify the activation of upstream transcriptional regulators that could explain the observed gene expression changes. IPA Canonical Pathways Analysis was performed to identify the activation of pathways from a library of canonical pathways.

### Immunohistochemistry for TNFα

Immunohistochemical staining was performed on colon tissue sections from control and irradiated animals with avidin–biotin complex (ABC) technique, diaminobenzidine (DAB) chromogen, and hematoxylin counterstaining. Appropriate positive and negative controls were included. The primary antibody was rabbit polyclonal antibody to TNFα (1:100) (Abcam, Cambridge, MA). The dilution of the second antibody (goat anti-rabbit) was 1:400.

### Statistical analysis

#### GeneSpring analysis

The expression profiles of unirradiated control samples from jejunum, ileum and colon were used as the baseline for generating the list of altered genes at each time point after TBI. Lists of gene were generated by *t*-test and Benjamini-Hochberg multiple testing correction with a false-discovery rate < 0.05. Significantly changed genes were selected with a cut-off of P < 0.05 and fold change ≥ 2.0.

#### IPA analysis

The significance of the association between the data set and the function/canonical pathway was determined by the P-value, which was calculated by Fisher’s exact test and Benjamini-Hochberg multiple testing correction. A P-value < 0.05 indicated a statistically significant, nonrandom association. Activation Z-score was used to identify the biological function/pathway expected to be increased or decreased. Z-score was also used to identify transcription factors that were significantly activated or inhibited. An absolute z-score of ≥ 2 was considered significant. Increased (activated) biological functions/pathways were identified by a z-score ≥ 2, and decreased (inhibited) functions/pathways by a z-score < −2.

## Availability of supporting data

The raw and normalized microarray data is available in Gene Expression Omnibus (GEO) with an accession number GSE74110 (https://urldefense.proofpoint.com/v2/url?u=http-3A__www.ncbi.nlm.nih.gov_geo_query_acc.cgi-3Facc-3DGSE74110&d=BQIEAg&c=6vgNTiRn9_pqCD9hKx9JgXN1VapJQ8JVoF8oWH1AgfQ&r=W8LxzO8ZOKsC96Kr1NIe4x1i6OCs2ec2O9G-iXxNmcI&m=MlqtHsTLnRF0Q6kFosq7gSr_vwvAm7tRBtRJghcCTGE&s=xphwihznMe0Amr6MuXesIsMdlrBputIhKWDEjR5LrCg&e=).

## Additional files

Additional file 1: Table S1. An excel file containing 18 lists of genes altered ≥ 2.0-Fold from jejunum, ileum and colon at days 4, 7 and 12 after 6.7-Gy and 7.4-Gy TBI including fold-change and *P*-values. (XLS 3319 kb) Additional file 2: Table S2. Altered genes regulating granulocyte adhesion and diapedesis in jejunum, ileum and colon at days 4, 7 and 12 after 6.7 Gy and 7.4 Gy TBI. (PDF 127 kb)

## Abbreviations

GI: Gastrointestinal
TBI: Total body irradiation
TNFα: Tumor necrosis factor α
CLDN2: claudin 2
IPA: Ingenuity Pathway Analysis
AhR: Activation of aryl hydrocarbon receptor
ECM: Extracellular matrix
ABC: Avidin–biotin complex
DAB: Diaminobenzidine
IACUC: Institutional Animal Care and Use Committee
AAALAC: Association for Assessment and Accreditation of Laboratory Animal Care
BARDA: Biomedical Advanced Research and Development Authority
ASPR: Assistant Secretary for Preparedness and Response
