# Supplementary material for: Gene expression profiling in non-human primate jejunum, ileum and colon after total-body irradiation: a comparative study of segment-specific molecular and cellular responses
Source: BMC Genomics. 2015 Nov 21;16:984. doi: 10.1186/s12864-015-2168-y (PMC4654820; doi:10.1186/s12864-015-2168-y)
Supplement: Additional file 2: Table S2. — Altered genes regulating granulocyte adhesion and diapedesis in jejunum, ileum and colon at days 4, 7 and 12 after 6.7 Gy and 7.4 Gy TBI. (PDF 127 kb) [file 12864_2015_2168_MOESM2_ESM.pdf]

Table S2. Altered genes regulating granulocyte adhesion and diapedesis in jejunum, ileum and colon at days 4, 7 and 12 after 6.7 Gy and 7.4 Gy TBI.

Symbol	Jejunum						Ileum						Colon					
	A1	A2	A3	A4	A5	A6	A1	A2	A3	A4	A5	A6	A1	A2	A3	A4	A5	A6
CCL2	↑5.54			↑6.37	↑2.70		↑3.29			↑3.67		↑2.21	↑3.82	↑2.45		↑5.32		
CCL3	↑3.34			↑4.18									↑2.22			↑3.26		↑2.04
CCL8		↑2.69	↑3.95			↑3.00								↑3.25		↑3.40		
CCL11													↑2.88			↑3.41		
CCL17																↑3.80		
CCL18				↑2.33									↓-2.39	↓-3.46		↓-2.88		
CCL19	↑13.21			↑12.84			↑7.10	↑2.78		↑5.26						↑5.70		
CCL20	↑6.63		↑8.58										↑9.16	↑3.07		↑11.51	↑4.51	
CCL21	↑2.99			↑3.20			↑2.49			↑2.35								
CCL23													↑16.50			↑31.68		
CCL24																↓-2.21		
CCL26																↑2.80		
CDH5				↑2.15												↑2.05		
CLDN1													↑2.08			↑2.35		
CLDN2													↑105.58	↑13.85		↑195.71		
CLDN3																↓-2.77		
CLDN4																↓-2.29		
CLDN7																↓-2.16		
CLDN8																↓-19.54		
CSF3R													↑2.58			↑3.13		
CXCL1	↑14.69			↑6.28						↑4.17			↑13.66	↑3.04		↑18.31		
CXCL2	↑8.67			↑10.45									↑15.03	↑4.80		↑20.20		
CXCL3	↑2.89									↑2.32			↑8.12	↑2.19		↑10.90		
CXCL6													↑2.77			↑2.65		
CXCL8				↑7.11						↑3.96			↑60.90	↑11.78	↑3.01	↑55.79		↑8.20
CXCL10	↑5.07	↑6.02				↑9.97				↑3.83		↑10.95	↑4.15			↑13.61		
CXCL11	↑4.00								↑3.622	↑3.83	↑2.94	↑10.14	↑9.53			↑33.63		
CXCL16													↑3.31			↑4.52		
CXCL17													↑12.20			↑31.43		
IL37		↓-3.42			↓-5.68													
IL1A													↑13.52	↑5.08		↑34.03		↑3.04
IL1B																↑4.77		
ITGA2																↑3.00		
ITGA6																↑2.19		
MMP2	↑2.47			↑2.87			↑2.30			↑3.13			↑2.61			↑2.92		
MMP3																↑2.45		
MMP7													↑10.94			↑41.21		
MMP9													↑4.55			↑2.86		
MMP15																↓-2.10		
MMP28																↓-2.11		
MSN		↓-2.11	↓-2.12		↓-2.29	↓-2.60			↓-2.36		↓-2.03			↓-2.12	↓-2.62			↓-3.51
PF4									↓-4.85			↓-5.77	↑9.52			↑13.76		
SDC3	↑2.08												↑2.42			↑3.06		
SELL																↑4.06		
SELP																↑2.09		
TNF																↑2.40		
TNFRSF11B	↑2.59			↑2.31									↑2.98			↑2.70		
VCAM1	↑2.55			↑2.99									↑2.64			↑2.54		
